# Associations between macular retinal vasculature and severity of idiopathic epiretinal membrane

**DOI:** 10.1186/s12886-023-02945-x

**Published:** 2023-05-05

**Authors:** Yun Hsia, Yi-Ting Hsieh

**Affiliations:** 1 Department of Ophthalmology, National Taiwan University Hospital Hsin-Chu Branch, Hsinchu, Taiwan; 2 Department of Ophthalmology, National Taiwan University Hospital, No 7, Chung-Shan S. Rd, Taipei, 100 Taiwan

**Keywords:** Epiretinal membrane, Optical coherence tomography angiography, Optical coherence tomography, Macular vasculature

## Abstract

**Background:**

To demonstrate the associations between the morphology of macular retinal vasculature and disease severity of idiopathic epiretinal membrane (ERM).

**Methods:**

Macular structures were assessed using optical coherence tomography (OCT), and were classified as “with pseudohole” or “without pseudohole”. The 3 × 3 mm macular OCT angiography images were analyzed using the Fiji software to obtain the vessel density, skeleton density, average vessel diameter, vessel tortuosity, fractal dimension, and foveal avascular zone (FAZ)-related parameters. The correlations between these parameters and ERM grading as well as visual acuity were analyzed.

**Results:**

For ERM with or without a pseudohole, increased average vessel diameter, decreased skeleton density, and decreased vessel tortuosity were all associated with inner retinal folding and thickened inner nuclear layer, indicating more severe ERM. In 191 eyes without a pseudohole, the average vessel diameter increased, fractal dimension decreased and vessel tortuosity decreased with increasing ERM severity. The FAZ was not associated with ERM severity. Decreased skeleton density (*r* = -0.37), vessel tortuosity (*r* = -0.35), and increased average vessel diameter (*r* = 0.42) were correlated with worse visual acuity (All *P* < 0.001). In 58 eyes with pseudoholes, a larger FAZ was associated with a smaller average vessel diameter (*r* = -0.43, *P* = 0.015), higher skeleton density (*r* = 0.49, *P* < 0.001), and vessel tortuosity (*r* = 0.32, *P* = 0.015). However, none of the retinal vasculature parameters correlated with visual acuity and central foveal thickness.

**Conclusion:**

Increased average vessel diameter, decreased skeleton density, decreased fractal dimension and decreased vessel tortuosity were good indicators of ERM severity and associated visual impairment.

## Background

Idiopathic epiretinal membrane (ERM) is characterized by fibrocellular proliferation on the inner surface of the retina [1, 2]. It can cause alteration in the retinal structures and may result in loss of visual acuity and metamorphopsia [1, 2]. The advent of optical coherence tomography (OCT) enables the visualization of changes in inner and outer retinal structures. Several specific biomarkers have been identified to depict the features of ERM, including ectopic inner foveal layer [3], disorganization of retinal inner layers [4], cotton ball signs [5, 6], and the gap between the ERM and the retinal surface [7]. However, the OCT images used for analysis were mostly B-scan images, which could only represent one or two cross sections of the whole macula.

In fact, ERM not only causes significant retinal vessel displacement as observed by fundus photography [8, 9], but also leads to changes in macular retinal vasculature, as demonstrated by optical coherence tomography angiography (OCTA). Previous studies have demonstrated that eyes with idiopathic ERM had decreased superficial and deep vessel density at the macula [10, 11] but increased vessel density at the central subfield [12, 13] and a smaller area of the foveal avascular zone (FAZ) [14, 15] compared to healthy control subjects. Greater vascular changes were associated with greater central foveal thickness (CFT) and poor postoperative visual outcomes [10]. Most studies have investigated the OCTA parameters obtained automatically using built-in software including vessel density and FAZ. However, other OCTA parameters such as skeleton density, vessel diameter, vessel tortuosity, and fractal dimension could describe more characteristics of the vascular changes accompanied with the retinal fold and distortion in ERM, and they could only be analyzed after image processing for the en face OCTA images [16, 17]. These parameters have been evaluated in various retinal diseases and found to be associated with disease severity [16–20], but seldom discussed in idiopathic ERM. We thought that the en face OCTA images could reveal the conditions of the central macula which may not be shown in one or two cross-sectional B-scan OCT images.

In the present study, we aimed to describe retinal vasculature changes using OCTA in a large cohort of patients with idiopathic ERM. We analyzed the associations between these vascular parameters and disease severity, as assessed using visual acuity, CFT, and the ERM staging system. Additionally, we compared the features of retinal vasculature changes between patients with and without macular pseudoholes. Furthermore, the OCT biomarkers and clinical features that predicted vascular parameters were analyzed.

## Methods

Patients diagnosed with idiopathic ERM by Dr. Yi-Ting Hsieh at the National Taiwan University Hospital between January 2016 and December 2019 were retrospectively enrolled. Patients with retinopathy other than ERM, high myopia, opaque media, visually significant cataract (Lens Opacity Classification System III grades C2–5, P2–5, NC3-6, or NO3-6), and a history of intraocular surgery other than uncomplicated cataract surgery were excluded. Additionally, patients with secondary ERM due to previous surgery, retinal laser therapy, or ocular inflammation were excluded. All patients underwent detailed ophthalmic examinations including best-corrected visual acuity (BCVA), slit-lamp biomicroscopy, dilated fundus examination, OCT, and OCTA. If a patient had bilateral idiopathic ERM, only one eye was randomly selected. Institutional review board approval was obtained from the National Taiwan University Hospital for this study (201803097RINB), and the study adhered to the tenets of the Declaration of Helsinki. The requirement for informed consent was waived owing to the retrospective nature of this study.

### OCT measurement

All patients underwent spectral-domain OCT (RTVue RT-100, version 3.5; Optovue, Inc., Fremont, CA, USA) to obtain standard 10 mm vertical and horizontal scans centered at the fovea. The presence of ellipsoid zone disruption, external limiting membrane disruption, inner retinal folding [7], and loss of foveal depression were documented. The CFT was obtained from the retinal thickness map. The thickness of the segmented retinal layers, including the internal limiting membrane-outer plexiform layer (ILM-OPL), ganglion cell layer-inner plexiform layer (GC-IPL), inner nuclear layer (INL), OPL, outer nuclear layer-retinal pigment epithelium (ONL-RPE), and maximal retinal thickness (MRT), was measured using a manual caliber. Eyes were then classified as “with pseudohole” and “without pseudohole,” [21] and macular pseudohole was characterized by a deep foveal pit, verticalized edges, and thickened macula caused by ERM traction [22, 23]. Those without pseudoholes were further classified into three grades of ERM severity according to the staging system of ERM proposed by Govetto et al. [3]. Grade 1 and grade 2 corresponded to the stage 1 and stage 2 ERM, respectively, and grade 3 consisted of the stage 3 and stage 4 ERM. In summary, the grade 1 ERM was characterized by a preserved foveal pit and well-defined retinal layers. The grade 2 ERM was characterized by loss of foveal pit and well-defined retinal layers. The grade 3 ERM was defined as having an ectopic inner foveal layer, with or without disrupted retinal layers.

### OCTA Parameters

All patients underwent OCTA using the Optovue RTVue XR Avanti with the AngioVue OCTA system. Only scans with a scan quality indicator of ≥ 5 were eligible for further analysis. A 3 × 3 mm region centered on the fovea was scanned. FAZ parameters, including area, perimeter, and circularity, were measured using a built-in software. Other OCTA parameters were measured using Fiji software (version 2.0.0, National Institutes of Health, Bethesda, MD, USA) to process the en face angiography map of the whole inner retinal slab (ILM-OPL). The image processing procedures were as follows: First, the FAZ was marked and circularity was calculated using the “circularity” function. The original RGB images were converted to 8-bit grayscale images, and the “default” algorithm was used for auto thresholding. The vessel density was calculated as the area occupied by white pixels (blood vessels) divided by the entire area of the binarized image. Next, the “skeletonize” function was used to create a skeletonized image. Skeleton density was calculated as the area occupied by the white pixels (skeletonized blood vessels) divided by the entire area of the skeletonized image. The average vessel diameter was calculated as vessel density divided by skeleton density. The fractal dimension was calculated by the “box-counting” method to quantify the branching complexity [19, 24, 25]. Vessel tortuosity was calculated as an average of the ratio of geodesic distance and the Euclidean distance of each vessel branch [25].

### Statistical analysis

All statistical analyses were performed using the R software (version 4.0.3; R Foundation for Statistical Computing, Vienna, Austria). For descriptive statistics, means and standard deviations were calculated for parametric numerical data, and percentages were calculated for categorical variables. BCVA was converted to the logarithm of the minimum angle of resolution (logMAR) for statistical analysis. Analysis of variance was used to compare the clinical characteristics and OCTA parameters between the patients with different ERM grades. Scheffe’s test was used for post-hoc analyses. Pearson’s correlation was used to assess the relationships between vascular parameters and visual acuity, CFT, or FAZ area. Independent *t*-test and chi-squared test were used to compare the clinical characteristics between patients with and without pseudoholes. Univariate linear regression analysis was performed to analyze the association between OCT characteristics and OCTA biomarkers. *P* values < 0.05 were considered as statistically significant.

## Results

We examined 249 eyes of 249 patients (146 men and 103 women) with idiopathic ERM. Fifty-eight eyes (23.3%) showed macular pseudoholes. The remaining 191 eyes without pseudoholes were further classified into three groups according to ERM severity: 75, grade 1; 50, grade 2; and 66, grade 3.

### Pseudohole vs. no pseudohole

Table 1 shows a comparison of clinical characteristics between patients with and without pseudoholes. Patients with pseudoholes were younger and had better BCVA. The retinal thickness and FAZ parameters were not different between the two groups; however, the OCTA parameters were significantly different. Eyes with pseudoholes had a lower skeleton density, larger average vessel diameter, and lower vessel tortuosity. We further analyzed the correlation between the FAZ area and other OCTA parameters in eyes with pseudoholes and found that a larger FAZ area was associated with higher vessel density (*P* = 0.008, *r* = 0.34, 95% confidence interval [CI] = 0.09 ‒ 0.55), higher skeleton density (*P* < 0.001, *r* = 0.49, 95% CI = 0.26 ‒ 0.67), higher vessel tortuosity (*P* = 0.015, *r* = 0.32, 95% CI = 0.07 ‒ 0.53), and smaller average vessel diameter (*P* = 0.015, *r* = -0.43, 95% CI = -0.62 ‒ -0.20).

**Table 1:** Comparisons of clinical characteristics and OCTA parameters between ERM with and without psuedohole

	**No pseudohole**	**Pseudohole**	***P*-value**
Case number	191 (76.7%)	58 (23.3%)	
Age (years)	68.1 ± 8.2	64.3 ± 9.0	0.006^*^
Male (%)	113 (59.2)	33 (56.9)	0.877
LogMAR visual acuity	0.28 ± 0.27	0.20 ± 0.23	0.044^*^
Central foveal thickness (μm)	378 ± 101	384 ± 89	0.615
Maximal retinal thickness (μm)	414 ± 97	427 ± 91	0.377
OCTA parameters			
FAZ area (mm^2^)	0.175 ± 0.117	0.202 ± 0.146	0.181
FAZ perimeter (mm)	1.65 ± 0.58	1.72 ± 0.67	0.472
FAZ circularity	0.74 ± 0.14	0.76 ± 0.15	0.258
Vessel density (%)	41.9 ± 5.7	40.8 ± 5.3	0.191
Skeleton density (%)	18.7 ± 4.5	16.1 ± 4.1	< 0.001^*^
Average vessel diameter	2.33 ± 0.46	2.64 ± 0.46	< 0.001^*^
Fractal dimension	1.81 ± 0.05	1.81 ± 0.02	0.339
Vessel tortuosity	1.22 ± 0.06	1.19 ± 0.05	< 0.001^*^

*FAZ* Foveal avascular zone

^*^Significant* P*-values

### Correlations between OCTA Parameters and ERM Severity, CFT and BCVA

**Fig. 1:**
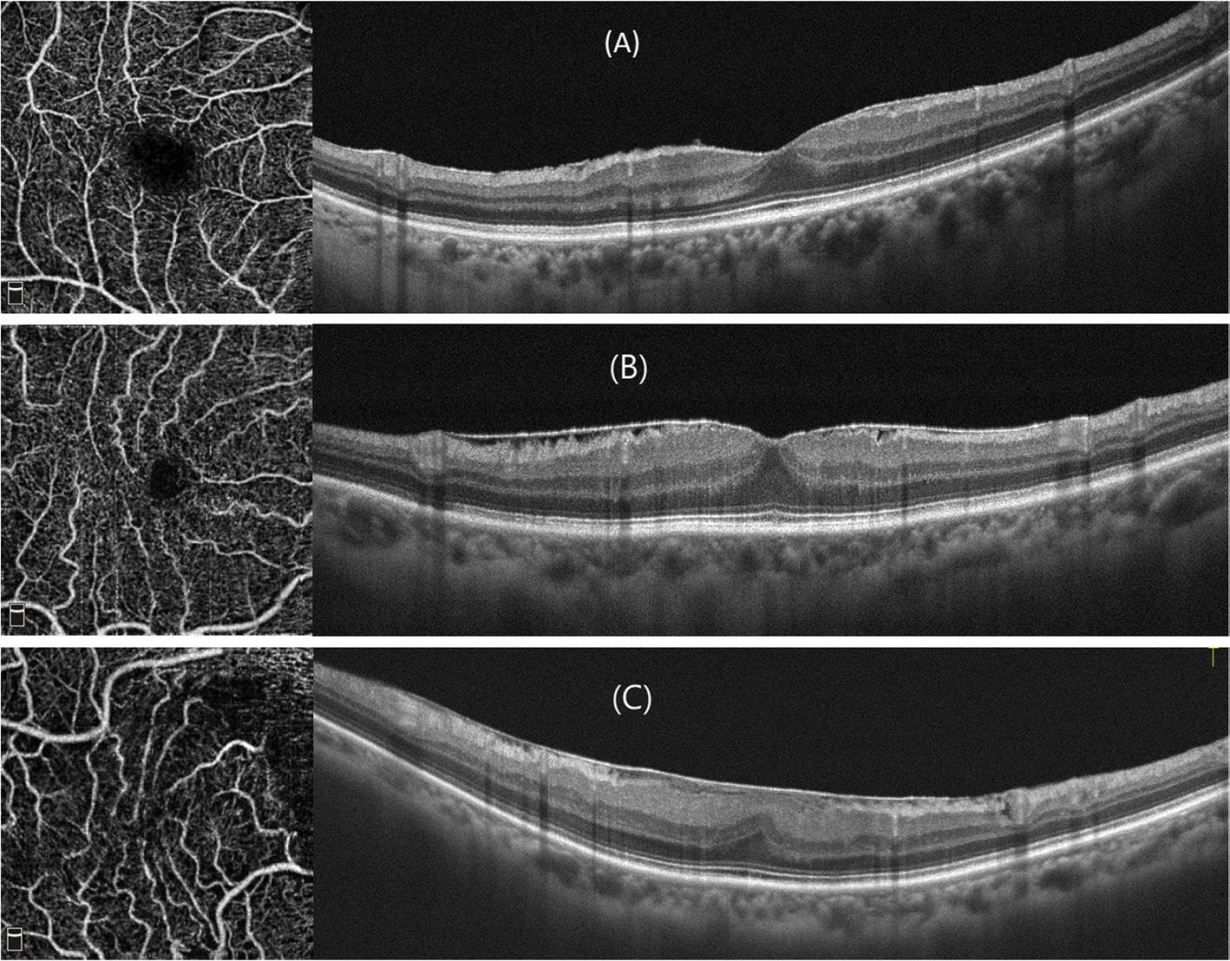
Optical coherence tomography and en face optical coherence tomography angiography of epiretinal membrane (ERM) with different disease severitiy. **A** Grade 1 ERM: Preservation of the foveal pit and well-defined retinal layers on the OCT. **B** Grade 2 ERM: Loss of the foveal pit but preservation of all retinal layers. **C** Grade 3 ERM: Ectopic inner foveal layer with organized retinal layers. As the grading of ERM increased, the skeleton density appeared to decrease, and the average vessel diameter increased

Table 2 shows the characteristics of the eyes without pseudoholes. Patients with more severe ERM grades had increased CFT and MRT. Eyes with grade 3 ERM had worse BCVA than those with grade 1 or 2 ERM. As the ERM severity increased, the skeleton density, fractal dimension, and vessel tortuosity decreased, whereas the average vessel diameter increased. The vessel density increased in grade 2, but then decreased in grade 3 when compared to grade 1 ERM. None of the FAZ parameters was associated with ERM severity. Figure 1 shows some examples of the OCTA and OCT of cases with different ERM severity. Table 3 shows the correlations between OCTA parameters, CFT, and visual acuity. For eyes without pseudoholes, a thicker CFT was associated with lower vessel density, skeleton density, fractal dimension, vessel tortuosity, and a larger average vessel diameter. Regarding BCVA, a larger logMAR, which indicates poorer vision, was associated with lower skeleton density, vessel tortuosity, and higher average vessel diameter. None of the FAZ parameters was associated with CFT or BCVA. For eyes with pseudoholes, none of the OCTA parameters were associated with CFT or BCVA.

**Table 2:** The clinical characteristics and OCTA parameters in patients with different severity of idiopathic epiretinal membrane

	**Grade 1** ^**a**^	**Grade 2** ^**a**^	**Grade** **3** ^**a**^	***P*** **-value**	**1 vs. 2**	**2 vs. 3**	**1 vs. 3**
Case number (%)	75 (39)	50 (26)	66 (35)				
Age (years)	67.1 ± 8.1	67.8 ± 8.8	69.3 ± 7.9	0.282	0.897	0.629	0.290
Male (%)	48 (64)	30 (60)	35 (53)	0.413			
LogMAR visual acuity	0.15 ± 0.17	0.22 ± 0.32	0.47 ± 0.23	< 0.001^*^	0.298	< 0.001^*^	< 0.001^*^
Central foveal thickness (μm)	291 ± 54	387 ± 51	470 ± 85	< 0.001^*^	< 0.001^*^	< 0.001^*^	< 0.001^*^
Maximal retinal thickness (μm)	353 ± 45	405 ± 59	491 ± 111	< 0.001^*^	< 0.001^*^	< 0.001^*^	< 0.001^*^
Parameters of optical coherence tomography angiography							
FAZ area (mm^2^)	0.170 ± 0.115	0.191 ± 0.125	0.168 ± 0.113	0.513	0.618	0.565	0.992
FAZ perimeter (mm)	1.64 ± 0.55	1.74 ± 0.60	1.60 ± 0.61	0.427	0.624	0.444	0.935
FAZ circularity	0.76 ± 0.12	0.73 ± 0.16	0.72 ± 0.14	0.507	0.565	0.893	0.236
Vessel density (%)	41.9 ± 5.2	43.5 ± 6.3	40.7 ± 5.6	0.030^*^	0.314	0.031^*^	0.427
Skeleton density (%)	20.8 ± 3.5	19.9 ± 4.3	15.3 ± 3.5	< 0.001^*^	0.416	< 0.001^*^	< 0.001^*^
Average vessel diameter	2.0 ± 0.1	2.2 ± 0.4	2.7 ± 0.4	< 0.001^*^	< 0.001^*^	< 0.001^*^	< 0.001^*^
Fractal dimension	1.81 ± 0.01	1.81 ± 0.01	1.80 ± 0.08	0.001^*^	0.887	0.006^*^	0.003^*^
Vessel tortuosity	1.24 ± 0.06	1.23 ± 0.06	1.18 ± 1.13	< 0.001^*^	< 0.001^*^	< 0.001^*^	< 0.001^*^

*FAZ* Foveal avascular zone

^*^Significant *P*-values

^a^Staging system proposed by Govetto et al. Grade 1 = stage 1; Grade 2 = stage 2; Grade 3 = stage 3 and 4

**Table 3:** The correlations between OCTA parameters and central foveal thickness and visual acuity

	**Central foveal thickness**				**LogMAR**			
	Without pseudohole		Pseudohole		Without pseudohole		Pseudohole	
	r (95% CI)	*P*-value	r (95% CI)	*P*-value	r (95% CI)	*P*-value	r (95% CI)	*P*-value
FAZ area (mm^2^)	-0.02 (-0.16 ~ 0.12)	0.777	0.02 (-0.24 ~ 0.28)	0.857	-0.09 (-0.22 ~ 0.06)	0.239	0.01 (-0.25 ~ 0.26)	0.957
FAZ perimeter (mm)	-0.03 (-0.17 ~ 0.11)	0.705	0.02 (-0.24 ~ 0.27)	0.904	-0.06 (-0.20 ~ 0.08)	0.404	-0.01 (-0.27 ~ 0.25)	0.939
FAZ circularity	-0.12 (-0.26 ~ 0.02)	0.096	0.07 (-0.19 ~ 0.32)	0.793	-0.06 (-0.20 ~ 0.09)	0.437	0.03 (-0.23 ~ 0.29)	0.807
Vessel density	-0.16 (-0.30 ~ -0.02)	0.023^*^	-0.07 (-0.32 ~ 0.19)	0.605	-0.09 (-0.23 ~ 0.06)	0.231	-0.13 (-0.37 ~ 0.14)	0.344
Skeleton density	-0.52 (-0.61 ~ -0.40)	< 0.001^*^	-0.10 (-0.35 ~ 0.16)	0.454	-0.37 (-0.48 ~ -0.24)	< 0.001^*^	0.04 (-0.22 ~ 0.30)	0.762
Vessel diameter	0.59 (0.49–0.68)	< 0.001^*^	0.10 (-0.17 ~ 0.35)	0.470	0.42 (0.30 ~ 0.53)	< 0.001^*^	-0.14 (-0.39 ~ 0.12)	0.288
Fractal dimension	-0.14 (-0.28 ~ -0.01)	0.050	-0.15 (-0.39 ~ 0.11)	0.262	-0.04 (-0.18 ~ 0.10)	0.566	0.02 (-0.24 ~ 0.27)	0.897
Vessel tortuosity	-0.48 (-0.58–0.35)	< 0.001^*^	-0.10 (-0.35 ~ 0.16)	0.463	-0.35 (-0.47 ~ -0.22)	< 0.001^*^	0.10 (-0.16 ~ 0.35)	0.440

*FAZ* Foveal avascular zone, *OCTA* Optical coherence tomography angiography

^*^Significant *P*-values

### Correlations between OCTA Parameters and OCT Characteristics

Table 4 shows the correlations between the OCT characteristics and OCTA parameters. Among the OCTA parameters, skeleton density, vessel tortuosity, and average vessel diameter were most correlated with OCT characteristics in ERM. Both decreased skeleton density, decreased vessel tortuosity, and increased average vessel diameter were associated with loss of foveal depression, presence of inner retinal folding, and increased inner retinal thickness. Decreased fractal dimension, indicating loss of branching complexity, was associated with increased inner retinal thickness and ELM disruption. Vessel density was negatively associated only with INL thickness. Decreased FAZ circularity was associated with loss of foveal depression and ellipsoid zone disruption. The FAZ area and perimeter were not associated with any of the OCT parameters analyzed.

**Table 4:** The analysis of correlations between OCT characteristics and OCTA parameters using univariable linear regression

	**FAZ area**		**FAZ perimeter**		**FAZ circularity**		**Vessel density**		**Skeleton density**		**Average vessel diameter**		**Fractal dimension**		**Vessel tortuosity**	
	*β (95% CI)*	*P*	*β (95% CI)*	*P*	*β (95% CI)*	*P*	*β (95% CI)*	*P*	*β (95% CI)*	*P*	*β (95% CI)*	*P*	*β (95% CI)*	*P*	*β (95% CI)*	*P*
Inner retinal folding^a^	-0.01 (-0.04 ~ 0.03)	0.715	-0.04 (-0.20 ~ 0.12)	0.606	-0.030 (-0.07 ~ 0.01)	0.108	-0.87 (-1.91 ~ 0.17)	0.099	-1.80 (-2.61 ~ 0.99)	< .001^*^	0.28 (0.16 ~ 0.40)	< .001^*^	-0.003 (-0.01 ~ 0.01)	0.590	-0.03 (-0.04 ~ -0.01)	< .001^*^
Foveal depression^b^	-0.02 (-0.05 ~ 0.02)	0.310	-0.07 (-0.22 ~ 0.08)	0.386	-0.04 (-0.08 ~ -0.01)	0.023^*^	-0.01 (-1.01 ~ 0.99)	0.987	-1.33 (-2.12 ~ -0.54)	0.001^*^	0.27 (0.15 ~ 0.38)	< .001^*^	-0.005 (-0.02 ~ 0.01)	0.382	-0.02 (-0.03 ~ -0.01)	0.005^*^
EZ disruption	-0.02 (-0.06 ~ 0.01)	0.155	-0.14 (-0.29 ~ 0.02)	0.082	-0.04 (-0.08 ~ 0.01)	0.020^*^	0.45 (-0.59 ~ 1.48)	0.395	-0.43 (-1.26 ~ 0.40)	0.309	0.10 (-0.02 ~ 0.22)	0.101	-0.003 (-0.01 ~ 0.01)	0.570	-0.01 (-0.02 ~ 0.01)	0.254
ELM disruption	-0.01 (-0.06 ~ 0.06)	0.881	-0.11 (-0.40 ~ 0.19)	0.479	-0.06 (-0.13 ~ 0.01)	0.091	-0.23 (-2.17 ~ 1.72)	0.820	-0.88 (-2.44 ~ 0.68)	0.267	0.17 (-0.06 ~ 0.40)	0.145	-0.04 (-0.06 ~ -0.01)	0.001^*^	-0.02 (-0.04 ~ 0.01)	0.337
GCIPLT (μm)^c^	-0.02 (-0.09 ~ 0.04)	0.530	-0.12 (-0.43 ~ 0.20)	0.465	0.02 (-0.05 ~ 0.10)	0.542	-0.93 (-3.02 ~ 1.15)	0.378	-1.64 (-3.31 ~ 0.02)	0.053	0.30 (0.03 ~ 0.52)	0.028^*^	-0.02 (-0.04 ~ 0.01)	0.172	-0.03 (-0.06 ~ -0.01)	0.004^*^
INLT (μm)^c^	0.03 (-0.03 ~ 0.08)	0.349	0.16 (-0.10 ~ 0.41)	0.235	-0.04 (-0.10 ~ 0.02)	0.216	-2.20 (-3.91 ~ 0.50)	0.012^*^	-2.19 (-3.55 ~ -0.83)	0.002^*^	0.26 (0.06 ~ 0.46)	0.013^*^	-0.05 (-0.07 ~ -0.04)	< .001^*^	-0.04 (-0.06 ~ -0.01)	0.004^*^
ILM_OPLT (μm)^c^	0.01 (-0.04 ~ 0.04)	0.851	0.03 (-0.17 ~ 0.22)	0.794	-0.001 (-0.05 ~ 0.04)	0.963	-0.98 (-2.27 ~ 0.32)	0.138	-1.62 (-2.65 ~ -0.59)	0.002^*^	0.27 (0.12 ~ 0.42)	< .001^*^	-0.02 (-0.04 ~ -0.01)	< .001^*^	-0.03 (-0.04 ~ -0.01)	0.006^*^
OPLT(μm)^c^	0.02 (-0.08 ~ 0.121)	0.677	0.09 (-0.40 ~ 0.57)	0.719	0.05 (-0.05 ~ 0.16)	0.387	-1.18 (-4.41 ~ 2.05)	0.473	-1.32 (-3.91 ~ 1.27)	0.317	0.20 (-0.19 ~ 0.58)	0.316	-0.01 (-0.04 ~ 0.03)	0.750	-0.02 (-0.07 ~ 0.02)	0.347
ONL_RPET (μm)^c^	0.001 (-0.05 ~ 0.05)	0.966	-0.04 (-0.28 ~ 0.20)	0.756	0.03 (-0.03 ~ 0.08)	0.311	-0.04 (-1.64 ~ 1.56)	0.959	0.03 (-1.26 ~ 1.31)	0.968	0.02 (-0.17 ~ 0.20)	0.876	-0.02 (-0.03 ~ 0.001)	0.068	-0.003 (-0.03 ~ 0.02)	0.775

*ELM* External limiting membrane, *EZ* Ellipsoid zone, *FAZ*, Foveal avascular zone, *GCIPL* Ganglion cell layer-inner plexiform layer, *ILM* Internal limiting membrane, *INL* Inner nuclear layer, *MRT* Maximal retinal thickness, *ONL* Outer nuclear layer, *OPL* Outer plexiform layer, *RPE* Retinal pigment epithelium, *T* Thickness

^*^Significant *P*-values

^a^absence of inner retinal folding served as the reference group

^b^preserved foveal depression served as the reference group

^c^ per 100 μm

## Discussion

Tangential traction of idiopathic ERM can cause significant changes in macular structure and retinal vasculature [10, 11, 13–15, 26–28]. The superficial capillary plexus (SCP) and deep capillary plexus (DCP) nourish the inner retina and are located in the layer of RNFL and retinal ganglion cell and in the junction between IPL and OPL, respectively [29]. The tractional force of idiopathic ERM from the retinal surface causes significant disorganization of the inner retinal structures and results in changes in these two capillary plexuses [4]. Several previous studies have investigated differences in vascular parameters between eyes with idiopathic ERM and their fellow eyes or healthy controls [10, 11, 13–15, 26–28]. In the present study, we further analyzed whether these vascular parameters may change with the severity of ERM assessed by the OCT-based staging system proposed by Govetto et al. [3] in which the ectopic inner foveal layer is the critical feature for grading. This ERM staging system exhibited clinical relevance by showing that visual acuity declined and CFT increased in more advanced stages [3]. In the present study, we not only confirmed the correlation between ERM severity and BCVA, but also found that the skeleton density decreased and the average vessel diameter increased proportionally with the severity of ERM. Since the tractional centripetal displacement of the inner retinal layer and Müller cell–driven proliferation simultaneously contribute to the development of the ectopic inner foveal layer in idiopathic ERM [3, 30], the changes in retinal vasculature may be more complicated than the simple result of contraction. Theoretically, the traction force of the ERM may damage retinal capillaries. In addition, as retinal folding and disorganization of retinal layers become more severe, the retinal capillary network becomes more difficult to detect. Furthermore, Kim et al. proposed that diminished microvasculature might be an artifact due to distorted retinal structure or stagnated blood flow caused by the compressive force of the ERM [11]. We hypothesized that the vascular network in eyes with severe ERM would be more difficult to be detected thoroughly, and therefore the vasculature shown in the en face OCTA would also be less complete. As a result, there is a decrease in the skeleton density measured using OCTA. Because large vessels are less affected and easier to detect, the average vessel diameter measured by OCTA will increase as ERM become worse. The findings that skeleton density decreased and average vessel diameter increased in the presence of inner retinal folding, loss of foveal depression, and increased INL thickness and CFT in this study further confirmed our proposal. Furthermore, these changes in the retinal vasculature correlated with worse visual acuity. Therefore, we believe that decreased skeletal density and increased average vessel diameter are good indicators of more severe ERM and functional deterioration.

Previous studies have shown that eyes with idiopathic ERM had lower vessel density in the SCP and DCP than in healthy controls [26], and the changes in vessel density correlated with visual acuity and CFT [10]. The centripetal tractional force led to the displacement of the capillary plexus toward the fovea. Therefore, the vessel density in the foveal region increased, the area of the FAZ decreased, and the vessel density of the parafoveal region decreased [10, 11, 13, 15, 26–28, 31]. In addition to the tractional force, pre-existing vascular insufficiency may also attribute to the reduction in blood flow before the development of ERM [26]. However, we found that vessel density was less significantly correlated with ERM severity and CFT. In ERM, large retinal vessels appear thicker in the presence of traction. Compensatory vessel dilatation may also occur in the presence of disturbed perfusion [19]. Kim et al. also proposed that the central displacement of larger retinal vessels and dilatation of stagnated capillaries may both contribute to the increasing vessel diameter and vessel density in more severe ERM [11]. These mixed effects of ERM on changes in vessel density may make it a less important indicator of ERM severity or visual impairment.

Fractal dimension is a measurement of the branching complexity of retinal vessels and usually decreases in myopia, glaucoma, and hypertension; however, it has shown conflicting results in diabetic retinopathy [32]. In the present study, the fractal dimension decreased significantly in grade 3 ERM. Kim et al. reported that the DCP had a decreased fractal dimension and higher lacunarity in more severe ERM stages [11]. They speculated that this finding was an artifact due to distorted retinal structure or stagnated blood flow caused by the compressive force of the ERM [11]. In the present study, decreased fractal dimension was associated with increased INL thickness. INL thickness is a biomarker that reflects the extent of tangential traction from ERM [33]. The abnormal traction force may cause damage to tiny capillaries, resulting in dropout and decreased fractal dimension. Since DCP supplied the INL, the changes in the INL may affect DCP more prominently, which is in line with the findings of Kim et al. [11].

A previous study found that eyes with ERM had more tortuous vessels than fellow eyes [34] and healthy controls [35] and were associated with more significant metamorphopsia [34]. In contrast, the present study demonstrated that the tortuosity of macular retinal vessels decreased in patients with more severe ERM. In addition, decreased vessel tortuosity was correlated with thicker CFT and worse BCVA. Theoretically, tangential traction from the ERM could cause prominent curling of the perifoveal capillary; alternatively, it could straighten and drag the surrounding retinal vessel toward the fovea [8, 36, 37]. The abnormally slow and fluctuated blood flow may cause the vulnerable and pulled capillary wall to become tortuous [16, 38]. However, our study results implied that the straightening effect of the strong tractional force might outweigh the curling effect in more severe ERM. Furthermore, as stated previously, the capillary network may be under detected due to retinal folding and disorganization of retinal layers in severe ERM; therefore, the measured vessel tortuosity could not reflect the presence of torturous capillaries.

Previous studies have shown that eyes with ERM have a smaller FAZ than healthy eyes [10, 11, 13, 15, 26–28, 31]. However, the present study found that the FAZ-related parameters had no association with ERM severity, OCT parameters, and visual acuity. Since the FAZ area is highly variable among individuals and could be affected by other factors, including CFT, axial length, sex, and choroidal thickness [28, 31, 39], direct comparison of FAZ among individuals may be biased. Furthermore, FAZ is largely different between fovea center-sparing ERM and fovea-attached ERM. Despite the similar pathogenesis, foveal center-sparing attachment may result in different morphological changes and clinical characteristics from fovea-attached ERM, including pseudoholes. In the present study, we classified patients according to the presence or absence of macular pseudoholes, which appeared as discrete, reddish, round, or oval lesions [23]. We found that eyes with pseudoholes had better visual acuity than those without pseudoholes. Hwang et al. reported that eyes with pseudoholes had similar visual acuity as those with mild ERM, but had better visual acuity than those with significant inner retinal thickening [21]. Since the eyes in our non-pseudohole group had ERM severity ranging from mild to severe, the overall visual acuity in this group may have been worse than that in the pseudohole group. Pierro et al. revealed that the FAZ area was smaller in eyes without pseudoholes than in those with pseudoholes owing to the stronger traction force [40]. In contrast, we did not find a significant difference in FAZ-related parameters between eyes with and without pseudoholes. The conflicting results between the present study and those of Pierro et al. could be explained by the different disease severities and individual variability of the FAZ. Macular pseudoholes with straight edges have the fovea as the epicenter of centripetal traction [22]. A more prominent centripetal force would lead to a shorter distance of the foveal edges and result in a smaller FAZ area. We found that a larger FAZ in eyes with pseudoholes was associated with a higher skeleton density, vessel tortuosity, and smaller vessel diameter. In summary, the FAZ may be a good indicator of ERM severity in comparison to healthy fellow eyes. However, it was less reliable to indicate the severity of ERM among different subjects, as it varied between individuals and with the diverse force direction of ERM. Instead, the skeleton density, vessel tortuosity, and vessel diameter correlated with OCT parameters and visual acuity, and could be regarded as a proxy of fovea-attached ERM severity.

This study has several limitations. First, we did not enroll healthy eyes; therefore, we could not investigate the differences in vascular parameters between healthy eyes and eyes with idiopathic ERM. However, the data provided by previous studies delineated the changes in vascular parameters in eyes with idiopathic ERM. Second, the present study focused on vascular changes in different types and severity of ERM. In the analysis of en face OCTA images, we chose the image from the retinal slab, which included both the SCP and DCP. However, previous studies have suggested that the changes in SCP and DCP may be distinct owing to the different locations and inherent characteristics of capillaries [11]. Third, vessel density is affected by several factors. Although we excluded patients with other types of retinopathy, high myopia, opaque media, systemic disease, and systemic vasoactive medication could have biased our results. Despite of the extensive use of skeletonized metrics from the en face OCTA in the literature, the concerns of low agreement between results obtained using different imaging processing methods and algorithms had been raised recently [41, 42]. Therefore, further studies are necessary to verify whether the findings in this study are consistent with those of other methods. Due to the retrospective nature of the present study, we did not have detailed documentation of metamorphopsia, which may be associated with changes in the macular retinal vasculature. Lastly, the enrollment of severe ERM with disorganized retinal layers was relatively difficult due to the poorer image quality and small case numbers. This may limit the analysis of microvascular changes and their correlations with visual impairment in this subgroup of patients.

In conclusion, the present study investigated the morphology of macular retinal vasculature in idiopathic ERM of different types and severity. Eyes with more severe fovea-attached ERM had decreased skeleton density, a larger average vessel diameter, and decreased vessel tortuosity, which was also associated with worse visual acuity and increased CFT. In eyes with pseudoholes, smaller FAZ were associated with decreased skeleton density, larger vessel diameter, and decreased vessel tortuosity. Both skeleton density and average vessel diameter were significantly correlated with loss of foveal pit depression, inner retinal folding, and a thicker INL, all of which are characteristics of severe ERM. Based on these findings, we propose that skeleton density, vessel tortuosity, and average vessel diameter could be potential indicators of disease severity and strength of the centripetal tractional force in idiopathic ERM. Future studies could focus on the changes in these parameters in post-operative eyes and their role in predicting visual outcomes.

## Data Availability

Data will be available under reasonable request to the corresponding author.

## Abbreviations

BCVA: Best-corrected visual acuity
CFT: Central foveal thickness
CI: Confidence interval
DCP: Deep capillary plexus
ERM: Epiretinal membrane
FAZ: Foveal avascular zone
GC-IPL: Ganglion cell layer-inner plexiform layer
INL: Inner nuclear layer
ILM-OPL: Internal limiting membrane-outer plexiform layer
logMAR: Logarithm of the minimum angle of resolution
MRT: Maximal retinal thickness
OCT: Optical coherence tomography
OCTA: Optical coherence tomography angiography
ONL-RPE: Outer nuclear layer-retinal pigment epithelium
SCP: Superficial capillary plexus

## References

[1] Stevenson W, Prospero Ponce CM, Agarwal DR, Gelman R, Christoforidis JB (2016). Epiretinal membrane: optical coherence tomography-based diagnosis and classification. Clin Ophthalmol.

[2] Bu SC, Kuijer R, Li XR, Hooymans JM, Los LI (2014). Idiopathic epiretinal membrane. Retina.

[3] Govetto A, Lalane RA, Sarraf D, Figueroa MS, Hubschman JP (2017). Insights Into Epiretinal Membranes: Presence of Ectopic Inner Foveal Layers and a New Optical Coherence Tomography Staging Scheme. Am J Ophthalmol.

[4] Zur D, Iglicki M, Feldinger L, Schwartz S, Goldstein M, Loewenstein A, Barak A (2018). Disorganization of Retinal Inner Layers as a Biomarker for Idiopathic Epiretinal Membrane After Macular Surgery-The DREAM Study. Am J Ophthalmol.

[5] Ortoli M, Blanco-Garavito R, Blautain B, Mastorakos N, Souied EH, Glacet-Bernard A (2021). Prognostic factors of idiopathic epiretinal membrane surgery and evolution of alterations of the central cone bouquet. Graefes Arch Clin Exp Ophthalmol.

[6] Tsunoda K, Watanabe K, Akiyama K, Usui T, Noda T (2012). Highly reflective foveal region in optical coherence tomography in eyes with vitreomacular traction or epiretinal membrane. Ophthalmology.

[7] Murase A, Asaoka R, Inoue T, Nagura K, Arasaki R, Nakamura K, Okawa K, Tanaka S, Yanagi Y, Maruyama-Inoue M, Kadonosono K (2021). Relationship Between Optical Coherence Tomography Parameter and Visual Function in Eyes With Epiretinal Membrane. Invest Ophthalmol Vis Sci.

[8] Dell'omo R, Cifariello F, Dell'omo E, De Lena A, Di Iorio R, Filippelli M, Costagliola C (2013). Influence of retinal vessel printings on metamorphopsia and retinal architectural abnormalities in eyes with idiopathic macular epiretinal membrane. Invest Ophthalmol Vis Sci.

[9] Kofod M, la Cour M (2012). Quantification of retinal tangential movement in epiretinal membranes. Ophthalmology.

[10] Kim YJ, Kim S, Lee JY, Kim JG, Yoon YH (2018). Macular capillary plexuses after epiretinal membrane surgery: an optical coherence tomography angiography study. Br J Ophthalmol.

[11] Kim J, Park KH (2021). TEMPORAL CHANGES OF PARAFOVEAL MICROVASCULATURE AFTER EPIRETINAL MEMBRANE SURGERY: An Optical Coherence Tomography Angiography Study. Retina.

[12] Cho KH, Park SJ, Cho JH, Woo SJ, Park KH (2016). Inner-Retinal Irregularity Index Predicts Postoperative Visual Prognosis in Idiopathic Epiretinal Membrane. Am J Ophthalmol.

[13] Nelis P, Alten F, Clemens CR, Heiduschka P, Eter N (2017). Quantification of changes in foveal capillary architecture caused by idiopathic epiretinal membrane using OCT angiography. Graefes Arch Clin Exp Ophthalmol.

[14] Isik-Ericek P, Sizmaz S, Esen E, Demircan N (2021). The effect of epiretinal membrane surgery on macular microvasculature: an optical coherence tomography angiography study. Int Ophthalmol.

[15] Okawa Y, Maruko I, Kawai M, Hasegawa T, Arakawa H, Iida T (2019). Foveal structure and vasculature in eyes with idiopathic epiretinal membrane. PloS one..

[16] Lee H, Lee M, Chung H, Kim HC (2018). QUANTIFICATION OF RETINAL VESSEL TORTUOSITY IN DIABETIC RETINOPATHY USING OPTICAL COHERENCE TOMOGRAPHY ANGIOGRAPHY. Retina (Philadelphia, Pa).

[17] Kim AY, Chu Z, Shahidzadeh A, Wang RK, Puliafito CA, Kashani AH (2016). Quantifying Microvascular Density and Morphology in Diabetic Retinopathy Using Spectral-Domain Optical Coherence Tomography Angiography. Invest Ophthalmol Vis Sci..

[18] Koulisis N, Kim AY, Chu Z, Shahidzadeh A, Burkemper B, Olmos de Koo LC, Moshfeghi AA, Ameri H, Puliafito CA, Isozaki VL, Wang RK, Kashani AH (2017). Quantitative microvascular analysis of retinal venous occlusions by spectral domain optical coherence tomography angiography. PloS one..

[19] Hsiao CC, Yang CM, Yang CH, Ho TC, Lai TT, Hsieh YT (2020). Correlations between visual acuity and macular microvasculature quantified with optical coherence tomography angiography in diabetic macular oedema. Eye.

[20] Hsia Y, Wang SW, Huang CJ, Hung KC, Chen MS, Ho TC (2021). Clinical Characteristics of Highly Myopic Patients With Asymmetric Myopic Atrophic Maculopathy-Analysis Using Multimodal Imaging. Invest Ophthalmol Vis Sci.

[21] Hwang JU, Sohn J, Moon BG, Joe SG, Lee JY, Kim JG, Yoon YH (2012). Assessment of macular function for idiopathic epiretinal membranes classified by spectral-domain optical coherence tomography. Invest Ophthalmol Vis Sci.

[22] Gaudric A, Aloulou Y, Tadayoni R, Massin P (2013). Macular pseudoholes with lamellar cleavage of their edge remain pseudoholes. Am J Ophthalmol..

[23] Hubschman JP, Govetto A, Spaide RF, Schumann R, Steel D, Figueroa MS, Sebag J, Gaudric A, Staurenghi G, Haritoglou C, Kadonosono K, Thompson JT, Chang S, Bottoni F, Tadayoni R (2020). Optical coherence tomography-based consensus definition for lamellar macular hole. Br J Ophthalmol.

[24] Zahid S, Dolz-Marco R, Freund KB, Balaratnasingam C, Dansingani K, Gilani F, Mehta N, Young E, Klifto MR, Chae B, Yannuzzi LA, Young JA (2016). Fractal Dimensional Analysis of Optical Coherence Tomography Angiography in Eyes With Diabetic Retinopathy. Invest Ophthalmol Vis Sci.

[25] Alam M, Thapa D, Lim JI, Cao D, Yao X (2017). Quantitative characteristics of sickle cell retinopathy in optical coherence tomography angiography. Biomed Opt Express.

[26] Coppe AM, Lapucci G, Gilardi M, Petruzzella F, Ripandelli G (2020). ALTERATIONS OF MACULAR BLOOD FLOW IN SUPERFICIAL AND DEEP CAPILLARY PLEXUSES IN THE FELLOW AND AFFECTED EYES OF PATIENTS WITH UNILATERAL IDIOPATHIC EPIRETINAL MEMBRANE. Retina.

[27] Mao J, Xu Z, Lao J, Chen Y, Xu X, Wu S, Zheng Z, Liu B, Shen L (2021). Assessment of macular microvasculature features before and after vitrectomy in the idiopathic macular epiretinal membrane using a grading system: An optical coherence tomography angiography study. Acta Ophthalmol.

[28] Hirata A, Nakada H, Mine K, Masumoto M, Sato T, Hayashi K (2019). Relationship between the morphology of the foveal avascular zone and the degree of aniseikonia before and after vitrectomy in patients with unilateral epiretinal membrane. Graefes Arch Clin Exp Ophthalmol.

[29] Coscas F, Sellam A, Glacet-Bernard A, Jung C, Goudot M, Miere A, Souied EH (2016). Normative Data for Vascular Density in Superficial and Deep Capillary Plexuses of Healthy Adults Assessed by Optical Coherence Tomography Angiography. Invest Ophthalmol Vis Sci..

[30] Lindqvist N, Liu Q, Zajadacz J, Franze K, Reichenbach A (2010). Retinal glial (Müller ) cells: sensing and responding to tissue stretch. Invest Ophthalmol Vis Sci.

[31] Kitagawa Y, Shimada H, Shinojima A, Nakashizuka H (2019). FOVEAL AVASCULAR ZONE AREA ANALYSIS USING OPTICAL COHERENCE TOMOGRAPHY ANGIOGRAPHY BEFORE AND AFTER IDIOPATHIC EPIRETINAL MEMBRANE SURGERY. Retina.

[32] Yu S, Lakshminarayanan V (2021). Fractal Dimension and Retinal Pathology: A Meta-Analysis. Appl Sci.

[33] Ichikawa Y, Imamura Y, Ishida M (2018). Inner Nuclear Layer Thickness, a Biomarker of Metamorphopsia in Epiretinal Membrane, Correlates With Tangential Retinal Displacement. Am J Ophthalmol.

[34] Sekiryu H, Nakao S, Hayashi T, Kaizu Y, Wada I, Arima M, Ishikawa K, Higashijima N, Morooka K, Sonoda KH (2019). Vessel Tortuosity Measurement in Epiretinal Membrane in Optical Coherence Tomography Angiography. Invest Ophthalmol Vis Sci.

[35] Mastropasqua R, D'Aloisio R, Viggiano P, Borrelli E, Iafigliola C, Di Nicola M, Aharrh-Gnama A, Di Marzio G, Toto L, Mariotti C and Carpineto P (2019) Early retinal flow changes after vitreoretinal surgery in idiopathic epiretinal membrane using swept source optical coherence tomography angiography. J Clin Med 8 (12). 10.3390/jcm8122067 10.3390/jcm8122067 PMC6947278 31771299

[36] Mansour AM, Mansour HA, Arevalo JF (2014). Spontaneous release of epiretinal membrane in a young weight-lifting athlete by presumed central rupture and centrifugal pull. Clin Ophthalmol.

[37] Liu J, Qian Y, Yang S, Yan L, Wang Y, Gao M, Liu L, Xiao Y, Mo B, Liu W (2017). Pathophysiological correlations between fundus fluorescein angiography and optical coherence tomography results in patients with idiopathic epiretinal membranes. Exp Ther Med.

[38] Kadonosono K, Itoh N, Nomura E, Ohno S (1999). Capillary blood flow velocity in patients with idiopathic epiretinal membranes. Retina.

[39] Tan CS, Lim LW, Chow VS, Chay IW, Tan S, Cheong KX, Tan GT, Sadda SR (2016). Optical Coherence Tomography Angiography Evaluation of the Parafoveal Vasculature and Its Relationship With Ocular Factors. Invest Ophthalmol Vis Sci..

[40] Pierro L, Iuliano L, Marchese A, Arrigo A, Rabiolo A, Bandello F (2019). Reduced vascular perfusion density in idiopathic epiretinal membrane compared to macular pseudohole. Int Ophthalmol.

[41] Courtie E, Kale A, Capewell N, Liu X, Gilani A, Montesano G, Teussink M, Veenith T, Denniston AK, Blanch RJ (2022). Skeletonization reduces the reliability of OCTA retinal blood flow analyses. Invest Ophthalmol Vis Sci..

[42] Freedman IG, Li E, Hui L, Adelman RA, Nwanyanwu K, Wang JC (2022). The Impact of Image Processing Algorithms on Optical Coherence Tomography Angiography Metrics and Study Conclusions in Diabetic Retinopathy. Transl Vis Sci Technol.

